# Characteristics and prognostic analysis of cystic vestibular schwannomas: prolonged surgical duration with limited impact on neurological function

**DOI:** 10.3389/fonc.2025.1589941

**Published:** 2025-07-30

**Authors:** Xuanpeng Li, Hongying Cai, Huijun Gong, Zi’ang Wang, Jibo Lv

**Affiliations:** ^1^ Trauma Center, First Affiliated Hospital of Kunming Medical University, Kunming Medical University, Kunming, Yunnan, China; ^2^ Student Affairs Office, Southwest Forestry University, Kunming, Yunnan, China; ^3^ Department of Neurosurgery, The First People’s Hospital of Qujing, Affiliated to Kunming Medical University, Qujing, Yunnan, China

**Keywords:** cystic vestibular schwannomas, solid vestibular schwannomas, tumor resection duration, neurological outcomes, tumor volume

## Abstract

**Introduction:**

This study aims to evaluate the differences between cystic vestibular schwannomas (CVS) and solid vestibular schwannomas (SVS) with respect to imaging characteristics, surgical duration, gross total resection (GTR) rates, and postoperative neurological functional outcomes. The goal is to inform individualized surgical planning and perioperative management.

**Methods:**

A retrospective analysis was conducted on 273 patients who underwent surgery for vestibular schwannomas. Patient data were divided into CVS and SVS groups. Comparisons were made regarding tumor volume, surgical duration, GTR rates, and postoperative preservation of facial nerve function and hearing. Multivariate analysis was used to identify independent predictors for postoperative functional outcomes.

**Results:**

The findings revealed that CVS cases had larger tumor volumes and longer surgical durations (96.6 minutes vs. 87.5 minutes) compared to SVS. Additionally, the rates of postoperative facial nerve function preservation and hearing preservation were lower in the CVS group, while no significant difference was found in GTR rates between the two groups. Multivariate analysis identified tumor volume and patient age as independent predictors of postoperative functional outcomes, whereas cystic changes had a limited impact on prognosis.

**Discussion:**

These results underscore the importance of thorough preoperative assessment of tumor volume and cystic characteristics in vestibular schwannomas. Evaluating these factors can optimize individualized surgical strategies and perioperative management, with the aim of improving postoperative functional outcomes for patients.

## 1 Introduction

Vestibular schwannomas (VSs) represent the most common tumors in the cerebellopontine angle region, accounting for approximately 8% of intracranial tumors. They originate from abnormal myelination of Schwann cells in the vestibular branch of the eighth cranial nerve. Currently, surgical resection remains the primary treatment modality for large (Koos grade 3-4) and progressive tumors (1). Cystic degeneration is a common feature of VSs, occurring in approximately 10-20% of all cases, and this specific pathological manifestation is closely related to clinical decision-making (2). Although the precise pathophysiological mechanisms underlying cystic degeneration remain unclear, traditional views generally consider cystic vestibular schwannomas (CVSs) to be more aggressive compared to solid vestibular schwannomas (SVSs). This aggressiveness is characterized by shorter clinical histories, more challenging adhesions to the brainstem and surrounding neurovascular structures, and consequently poorer facial nerve outcomes, higher surgical complication rates, and increased mortality when Gross total tumor resection (GTR) is pursued (2–5). Therefore, the 2003 Vestibular Schwannoma Consensus Meeting recommended analyzing and reporting CVSs as a distinct subgroup of VSs (6). However, recent studies have presented differing perspectives. A 2012 systematic review indicated no significant differences between CVSs and SVSs regarding the extent of resection, surgical complications, and mortality, except for poorer facial nerve outcomes in CVSs (7). Furthermore, a 2024 multi-institutional cohort study suggested that the poorer facial nerve outcomes and lower GTR rates in CVSs patients might primarily result from larger tumor volumes rather than the presence of cystic components per se (8). These findings challenge previous surgical strategies for CVSs, which prioritized subtotal resection (STR) to preserve cranial nerve function (9), as patients undergoing STR undoubtedly experience higher tumor recurrence rates compared to those receiving GTR (10).

Recent improvements in surgical philosophy and intraoperative electrophysiological techniques have likely contributed to changes in CVSs surgical outcomes, enhancing neurovascular prognoses. Therefore, it is necessary to retrospectively analyze clinical characteristics of VSs treated surgically in recent years, comparing differences between CVSs and SVSs. Beyond previously emphasized outcome indicators, it is essential to consider additional objective metrics, such as tumor resection duration, GTR rates, and imaging characteristics. This study retrospectively analyzed data from patients with unilateral sporadic VSs who underwent tumor resection via the retrosigmoid approach, comparing CVSs and SVSs groups regarding demographic factors, imaging features, intraoperative tumor resection duration, GTR rates, and neurological outcomes, aiming to deepen understanding of CVSs characteristics and guide optimized treatment decisions.

## 2 Methods

### 2.1 Study design and patient selection

This single-center retrospective cohort study included consecutive patients who underwent tumor resection via the retrosigmoid approach at our institution between January 2018 and June 2024, with postoperative pathological confirmation of VSs. Inclusion criteria were: (1) primary tumor cases; (2) complete preoperative imaging and follow-up data. Exclusion criteria included: (1) genetic syndromes such as neurofibromatosis type 2 (NF2); (2) prior radiotherapy; (3) recurrent tumor cases. All patient data, including imaging, pathology reports, surgical records, and postoperative follow-up, were comprehensively stored in the hospital information system (HIS). The study was conducted in accordance with the Declaration of Helsinki, and the study protocol and a waiver of written informed consent were approved by the Clinical Research Center of First Affiliated Hospital of Kunming Medical University (study approval no.: 202312283) and the Ethics Committee of First Affiliated Hospital of Kunming Medical University (ethics approval no.: kmmu20231637).

### 2.2 Imaging assessment

**Figure 1:**
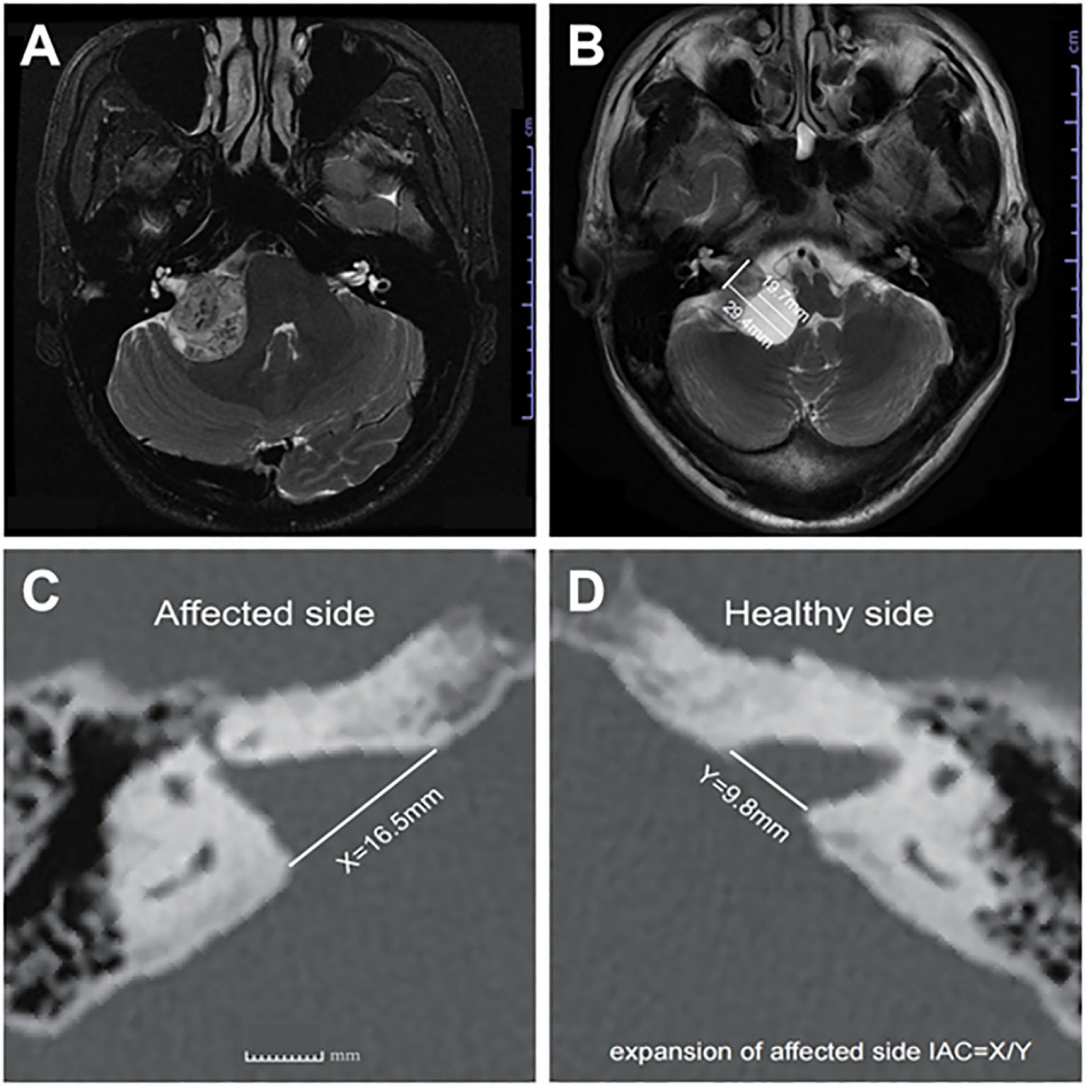
**(A)** Axial T2-weighted MRI image showing representative images of SVs, and **(B)** representative images of CVs, **(C)** shows the distance X from the affected side IAC nasal crest to the dorsal crest, and **(D)** the distance Y from the healthy side IAC nasal crest to the dorsal crest; X/Y = expansion of affected side IAC.

MRI images were independently and blindly evaluated by three senior specialists (one neuroradiologist, one neurologist, and one skull base neurosurgeon). CVS was defined radiologically as the presence of one or more cystic components on T2-weighted images, with cumulative cystic diameters exceeding 50% of the tumor’s maximum average diameter (**Figures 1A, B**) (11). Tumor grading followed the 2003 International Vestibular Schwannoma Consensus Meeting criteria: Grade 0 (purely intracanalicular), Grade 1 (1–10 mm), Grade 2 (11–20 mm), Grade 3 (21–30 mm), Grade 4 (31–40 mm), and Grade 5 (>40 mm) (6).

### 2.3 Clinical metrics quantification

- **Tumor resection duration**: Time from dural opening and tumor exposure to complete tumor removal (validated via surgical videos and anesthesia records).
- **Extent of resection**: Evaluated using the Goss classification based on enhanced MRI at 3 months postoperatively: Gross total resection (GTR): 100% resection; near-total resection (NTR): ≥98% resection; subtotal resection (STR): 95–98% resection; partial resection (PR): <95% resection (12).
- **Internal auditory canal (IAC) expansion index**: Measured via axial bone-window CT scans, calculating the ratio of distances between the nasal crest and dorsal crest of the affected versus healthy sides (**Figures 1C, D**) (13).

### 2.4 Functional assessment systems

- **Facial nerve function**: Assessed using the House-Brackmann (HB) grading system, with HB grades I-III defined as preserved function (14).
- **Hearing function**: Evaluated according to AAO-HNS guidelines, with pure-tone audiometry (PTA) and word recognition scores (WRS) measured preoperatively (1 week prior) and postoperatively (2 weeks after surgery). Practical hearing preservation was defined as AAO-HNS classes A-C (PTA <50 dB or WRS ≥50%) (15).

### 2.5 Statistical analysis

Data analysis was performed using SPSS (v26.0, IBM Corp., Chicago, USA). Continuous variables were tested for normality using the Shapiro-Wilk test; normally distributed data were expressed as mean ± standard deviation and compared using independent-sample t-tests. Non-normally distributed data were expressed as median (interquartile range) and compared using Mann-Whitney U tests. Categorical variables were described as frequencies (percentages) and compared using χ² tests or Fisher’s exact tests. Binary logistic regression models identified potential predictors, with variables showing P ≤ 0.05 in univariate analysis included in stepwise multivariate logistic regression models. The adjustment variables of the final model are all the selected variables except the target variable. Results were presented as P-values, odds ratios (OR), and 95% confidence intervals (CI). The forest map is visualized using R (version 4.4.4). Linear regression analyzed continuous dependent variables, with results expressed as P-values, regression coefficients (B values) and R². Statistical significance was set at P < 0.05.

## 3 Results

### 3.1 Cohort characteristics

The demographic characteristics and preoperative information of the patients are detailed in **Table 1**. A total of 273 patients were included in the study, with an average age of 48.5 ± 9.6 years (range: 22–73 years), of which 163 were female (59.7%). There were 127 cases of left-sided lesions (46.5%), and the average duration of symptoms (DOS) was 31.3 ± 18.0 months (range: 0.5–81 months). The average maximum tumor diameter was 32.1 ± 9.9 mm (range: 10.9 - 62.8 mm). According to the tumor grading criteria, there were 30 cases classified as grade 2 (11.0%), 82 cases as grade 3 (30.0%), 103 cases as grade 4 (37.7%), and 58 cases as grade 5 (21.2%). The most common clinical symptom was hearing loss in the same ear, with an incidence of 90.8% (248 cases), followed by tinnitus (69.6%, 190 cases) and vertigo (39.6%, 108 cases). The incidence of facial nerve paralysis was 5.5% (15 cases), including 13 cases classified as HB grade II and 2 cases as grade III.

**Table 1:** Patient demographics and preoperative characteristics.

Characteristic	Mean± SD (range) or No. (%)			P-value*
	Total (N=273)	cVS (N = 57)	sVS (N = 216)	
Age (year)		50.0 ± 8.4 (29-69)	48.1 ± 9.8 (22-73)	0.093
Female	163 (59.7)	33 (57.9)	130 (60.2)	0.098
Left Side	127 (46.5)	24 (42.1)	103 (47.7)	0.564
Symptoms at time of diagnosis				
Hypoacusis	248 (90.8)	54 (94.7)	194 (89.4)	0.375
Tinnitus	190 (69.6)	39 (68.4)	151 (69.6)	0.956
Vertigo	108 (39.6)	22 (38.6)	86 (39.8)	0.988
Facial nerve palsy	15 (5.5)	4 (7.0)	11 (5.1)	0.810
DOS (month)	31.3 ± 18.0 (0.5-81)	28.2 ± 19.0 (0.5-68)	32.1 ± 17.6 (0.5-81)	0.074
Maximum tumor diameter (mm)	32.1 ± 9.9 (10.9-62.8)	34.1 ± 9.5 (15.0-58.4)	31.6 ± 10.0 (10.9-62.8)	0.042
≤20	30 (11.0)	3 (3.5)	27 (12.5)	0.188
20-30	82 (30.0)	15 (26.3)	67 (31.0)	0.599
30-40	103 (37.7)	27 (47.4)	76 (35.2)	0.125
>40	58 (21.2)	12 (21.1)	46 (21.3)	1.000
Pre-op AAO-NHS classifcation				
Class A	48 (17.6)	6 (10.5)	42 (19.4)	0.168
Class B	87 (31.9)	13 (22.8)	75 (34.7)	0.120
Class C	56 (20.5)	13 (22.8)	43 (19.9)	0.766
Class D	82 (30.0)	25 (43.9)	57 (28.4)	0.012
Pre-op hearing preservation				
Preserved	191 (70.0)	32 (56.1)	159 (73.6)	0.012
PTA (dB)	42.3 ± 20.4 (10-86)	48.8 ± 22.2 (10-85)	40.9 ± 19.8 (10–86)	0.024
WRS (%)	73.7 ± 13.6 (50-100)	74.5 ± 13.7 (50-100)	73.6 ± 13.6 (50–100)	0.356
Not preserved	82 (30.0)	25 (43.9)	57 (26.4)	0.012
Expansion of affected side IAC	1.53 ± 0.42 (0.94-3.08)	1.63 ± 0.49 (0.98-3.08)	1.50 ± 0.40 (0.94-3.05)	0.016

DOS, duration of symptoms; Expansion of affected IAC = Diameter of affected side of IAC/Diameter of healthy side of IAC; *P-value shows the difference between CVs and SVs at the time of diagnosis.

### 3.2 CVs are associated with larger tumor size, greater IAC expansion, and lower rates of preoperative hearing preservation

In this study, there were 57 cases of CVSs, accounting for 20.9% of the total number of patients. When comparing demographic characteristics, including age, gender, and tumor laterality, no statistical differences were found between the CVSs and SVSs group. Additionally, no statistical differences were noted between the two groups regarding symptoms at the time of diagnosis and DOS. However, compared to the SVSs group, the CVSs group had significantly larger tumor volumes at the first visit, with maximum tumor diameters of 32.1 ± 17.6 mm and 28.2 ± 19.0 mm, respectively (P = 0.042). Additionally, the expansion of affected side IAC was significantly greater in the CVSs group compared to the SVSs group (1.63 ± 0.49 vs. 1.50 ± 0.40, P = 0.016). Notably, the proportion of patients in the CVSs group with hearing preservation at the time of diagnosis was significantly lower than that in the SVSs group (56.1% vs. 73.6%, P = 0.012) (**Table 1**).

**Figure 2:**
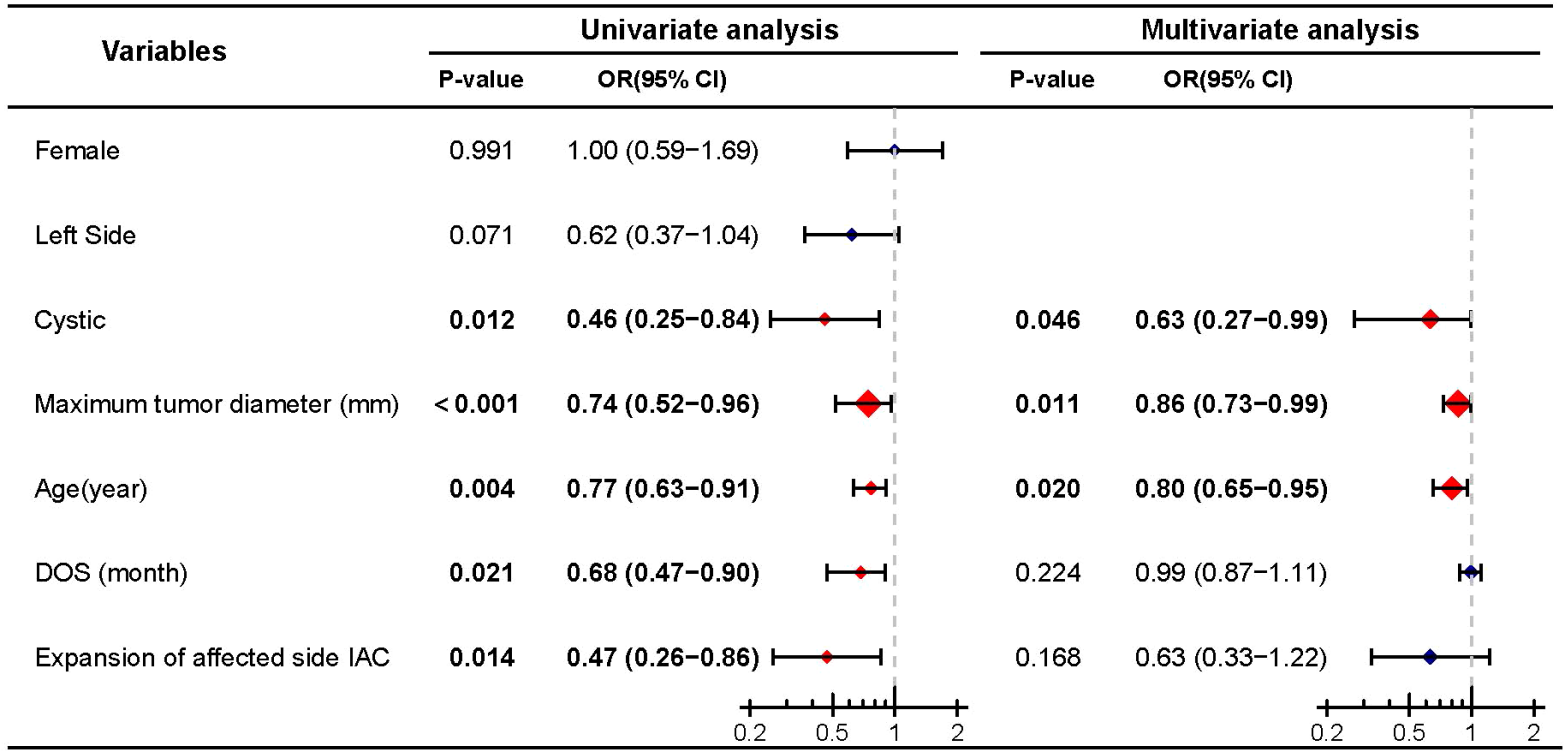
Univariate and multivariate analyses of preoperative hearing preservation. Significant differences (P < 0.05) are shown in bold and identified with red diamonds in the forest plot.

To further elucidate the relationship between cyst formation and preoperative hearing preservation, we conducted a stepwise multivariate binary logistic regression analysis on preoperative hearing preservation. Univariate analysis indicated that cystic (P = 0.012), maximum tumor diameter (P < 0.001), age (P = 0.004), DOS (P = 0.021), and expansion of affected side IAC (P = 0.014) were influencing factors for preoperative hearing preservation. Multivariate analysis revealed that cystic (P = 0.046), maximum tumor diameter (P = 0.011), and age (P = 0.020) were independent influencing factors for preoperative hearing preservation (**Figure 2**).

### 3.3 Postoperative outcome analysis

#### 3.3.1 CVS are associated with longer resection duration and lower rates of postoperative facial nerve function and hearing preservation

As shown in **Table 2**, the average duration of tumor resection for all patients was 89.4 ± 24.5 minutes. The proportion of patients with good facial nerve function postoperatively was 76.6% (209/273), and the overall postoperative hearing preservation rate was 52.7% (144/273). Notably, all but 7 patients underwent GTR (97.4%, 266/273). Inter-group comparison revealed no statistically significant difference in GTR rate between the CVSs and SVSs groups (96.5% vs. 97.6%, P=0.257). However, significant differences were observed between the CVS and SVSs groups in terms of tumor resection duration (96.6 ± 20.7 vs. 87.5 ± 25.1, P=0.006), the rate of good postoperative facial nerve function (64.9% vs. 79.6%, P=0.021), and postoperative hearing preservation rate (59.4% vs. 78.6%, P=0.024). Specifically, compared to the SVSs group, the CVSs group had a longer tumor resection duration, and significantly lower rates of good facial nerve function and hearing preservation.

**Table 2:** Summary of postoperative characteristics.

Characteristic	Mean± SD (range) or No. (%)			P-value*
	Total (N=273)	cVS (N = 57)	sVS (N = 216)	
GTR	266 (97.4)	55 (96.5)	211 (97.6)	0.257
Tumor resection duration (min)	89.4 ± 24.5 (35-160)	96.6 ± 20.7 (53-159)	87.5 ± 25.1 (35-160)	0.006
House-Brackmann grade				
Grade I-III	209 (76.6)	37 (64.9)	172 (79.6)	0.021
Grade IV-VI	64 (23.4)	20 (35.1)	44 (20.4)	0.021
Post-op AAO-NHS classifcation**				
Class A	21 (11.0)	0 (0.00)	21 (13.2)	<0.001
Class B	53 (27.7)	8 (25.0)	45 (28.3)	0.165
Class C	70 (36.6)	11 (34.3)	59 (37.1)	0.078
Class D	47 (24.6)	13 (40.6)	34 (21.4)	0.024
Post-op hearing preservation**				
Preserved	144 (75.4)	19 (59.4)	125 (78.6)	0.024
PTA (dB)	52.2 ± 21.9 (12-96)	58.7 ± 15.4 (36-85)	51.2 ± 22.5 (12-96)	0.080
WRS (%)	69.8 ± 12.1 (50-100)	64.4 ± 12.0 (50-95)	70.1 ± 12.1 (50-100)	0.180
Not preserved	47 (24.6)	13 (40.6)	34 (21.4)	0.024

GTR, Goss tumor resection; *P-value shows the difference between CVs and SVs at the time of diagnosis; **Total sample size is 191 cases, excluding 82 patients with preoperative hearing loss.

#### 3.3.2 Cystic degeneration and larger tumor size significantly prolong tumor resection duration

To further clarify the impact of cystic degeneration on tumor resection duration, we employed linear regression to analyze the relationship between known independent variables and tumor resection duration (dependent variable). The results showed that there was no significant collinearity among the 7 selected independent variables (VIF < 5), and the observations were mutually independent (D-W value = 1.77). The overall model explained a high proportion of the variance in tumor resection duration (R²=0.664, adjusted R²=0.655, F=74.933, P < 0.001). The analysis revealed that tumor size (B=1.996, P < 0.001) and cystic (B=4.780, P=0.031) had a significant impact on tumor resection duration. Specifically, for every 1 mm increase in the maximum diameter of the tumor, the resection duration was extended by an average of 1.996 minutes; in the presence of cystic, the resection duration was extended by an average of 4.78 minutes (**Table 3**).

**Table 3:** Results of linear regression analysis on tumor resection duration.

	Unstandardized Coefficient		Standardized Coefficient	t	P value	Collinearity Diagnosis	
	B	Standard error	Beta			Tolerance	VIF
(Constant)	30.868	6.037		5.113	0.000		
Female	-1.534	1.794	-0.031	-0.855	0.393	0.986	1.015
Left Side	-0.655	1.777	-0.013	-0.368	0.713	0.971	1.029
Cystic	4.780	2.204	0.079	2.169	0.031	0.951	1.051
Tumor maximum diameter (mm)	1.996	0.098	0.807	20.448	<0.001	0.813	1.231
Age (year)	-0.029	0.093	-0.011	-0.309	0.758	0.964	1.037
DOS (month)	0.048	0.052	0.035	0.927	0.355	0.872	1.146
Expansion of affected side IAC	-3.755	2.202	-0.065	-1.705	0.089	0.881	1.136
R^2^	0.664						
Adjusted R²	0.655						
F	F(7,265)=74.933,**P<0.001**						
D-W value	1.770						

#### 3.3.3 Tumor size and age are independent predictors of postoperative hearing preservation and facial nerve function

**Figure 3:**
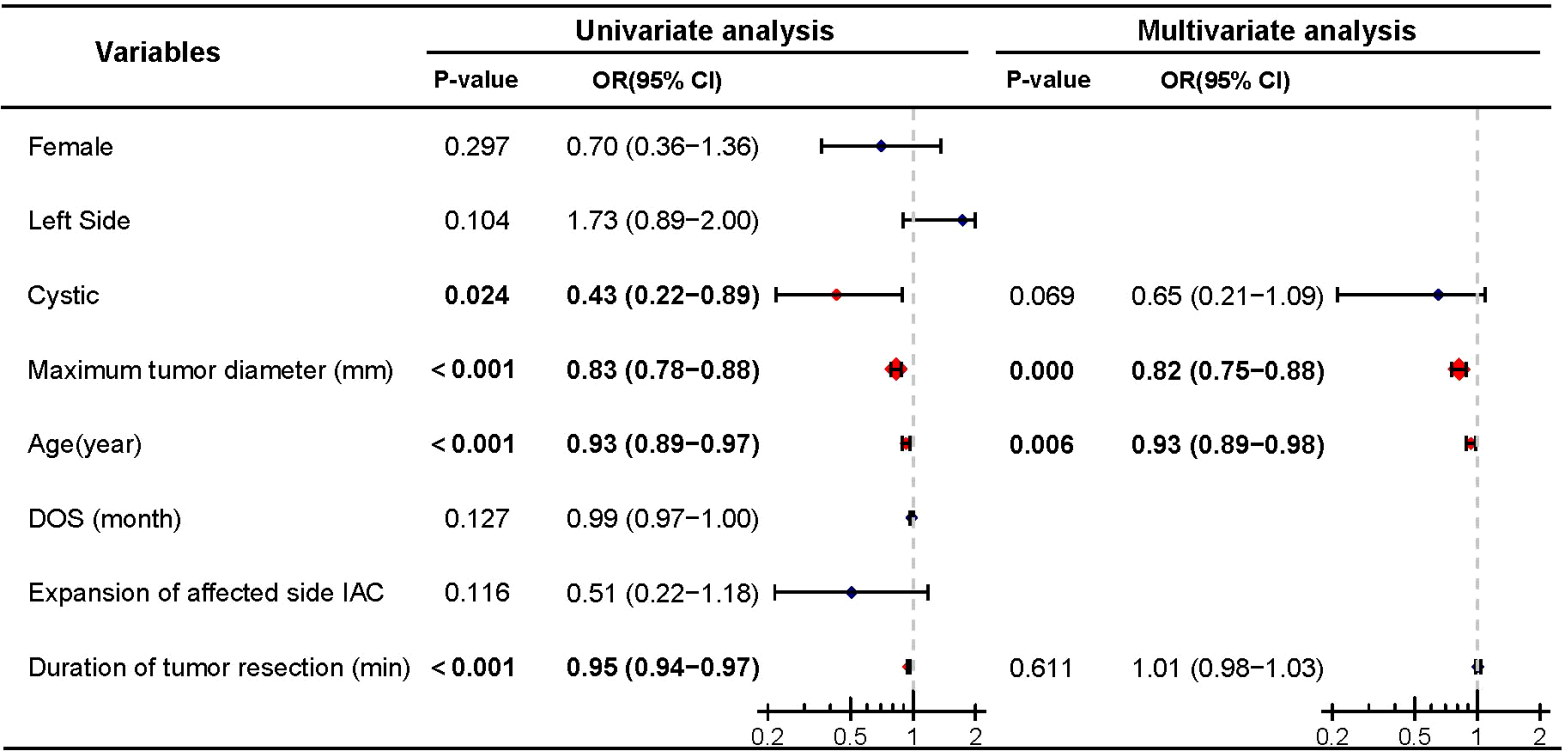
Univariate and multivariate analyses of postoperative hearing preservation. Significant differences (P < 0.05) are shown in bold and identified with red diamonds in the forest plot.

Among patients with preoperative hearing preservation, 75.4% (144/191) maintained hearing postoperatively. Of these, 21 patients (11.0%) were classified as Grade A according to the AAO-NHS classification, 53 patients (27.7%) as Grade B, and 70 patients (36.6%) as Grade C. Then, we conducted a Logistic regression analysis on postoperative hearing preservation (dependent variable) in patients with preoperative hearing preservation. Univariate analysis revealed that cystic (P=0.024), tumor maximum diameter (P<0.001), age (P<0.001), and duration of tumor resection (P<0.001) were significant predictors of postoperative hearing preservation. Further multivariate logistic regression analysis confirmed that only tumor maximum diameter (P<0.001) and age (P=0.001) were identified as independent predictive factors of postoperative hearing preservation, while cystic degeneration (P=0.069) and duration of tumor resection (P=0.611) did not demonstrate independent predictive value (**Figure 3**).

**Figure 4:**
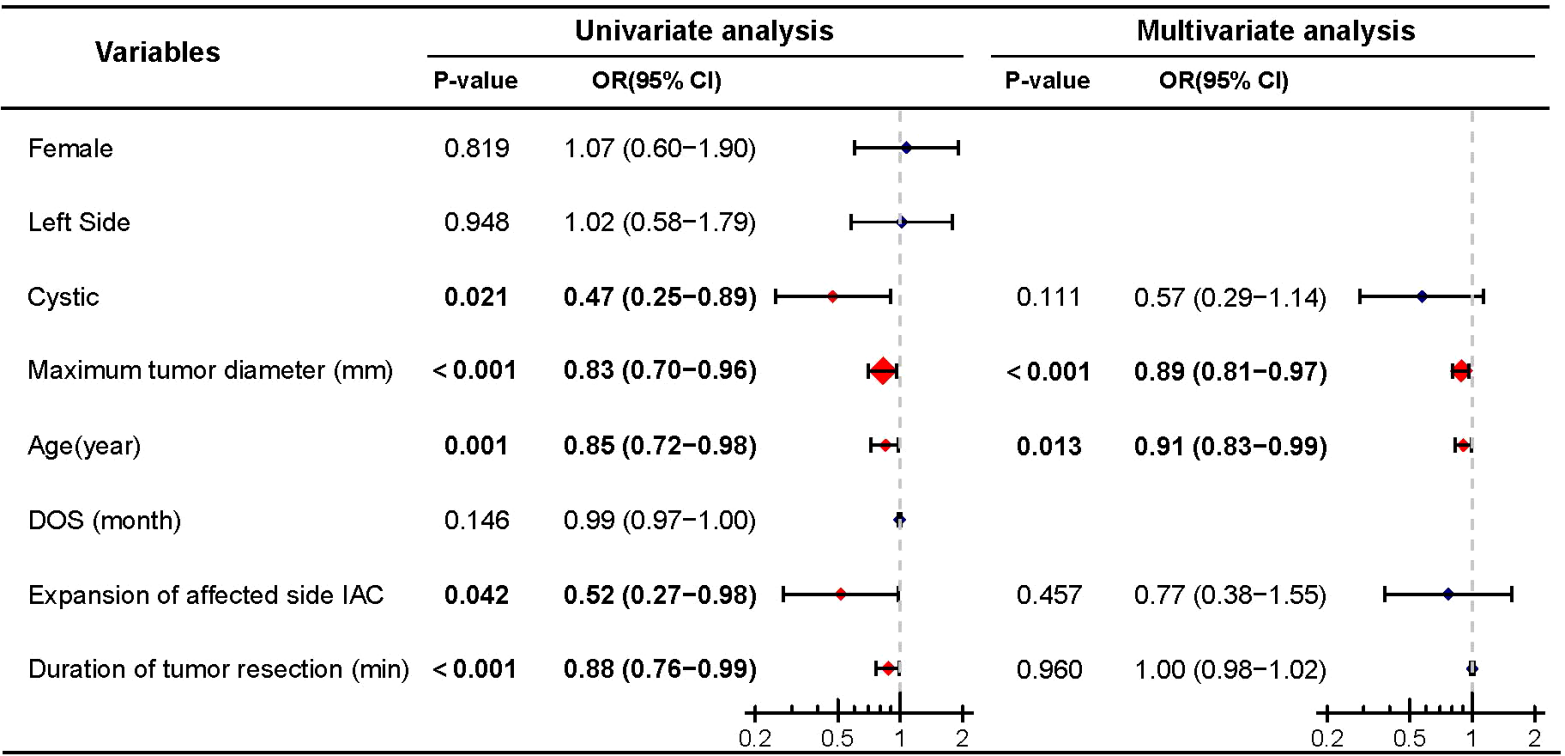
Univariate and multivariate analyses of postoperative good facial nerve function. Significant differences (P < 0.05) are shown in bold and identified with red diamonds in the forest plot.

Similarly, logistic regression analysis was performed to assess the impact of cystic degeneration on postoperative facial nerve function. Univariate analysis indicated that cystic (P=0.021), maximum tumor diameter (P<0.001), age (P=0.001), expansion of the affected side IAC (P=0.042), and the duration of tumor resection (P<0.001) were predictive factors for good postoperative facial nerve function. Further multivariate logistic regression analysis revealed that maximum tumor diameter (P<0.001) and age (P=0.013) were independent predictive factors for good postoperative facial nerve function, while cystic (P=0.069), expansion of the affected side IAC (P=0.457), and the duration of tumor resection P=0.960) were not included as independent predictive factors (**Figure 4**).

## 4 Discussion

Cystic degeneration is a common feature of VSs, accounting for approximately 10-20% of all VSs (2). Although the exact pathophysiological mechanism of cystic degeneration in VSs remains unclear, previous studies have suggested that CVSs are more aggressive than SVSs, with shorter clinical histories and poorer postoperative outcomes, including facial nerve function, surgical complications, and mortality rates, due to their invasive nature (2–5). However, recent studies have reported contradictory findings, indicating no significant differences in extent of resection, surgical complications, or mortality rates between CVSs and SVSs (7, 8). Therefore, we conducted a retrospective clinical analysis of VSs treated with surgical resection in recent years, focusing on a detailed comparison of the differences between CVSs and SVSs.

The results of this study indicate significant differences between CVSs and SVSs in tumor resection duration. Firstly, the presence of cysts is significantly correlated with longer tumor resection times. Linear regression analysis suggests that both tumor size and cystic degeneration can extend the duration of the surgical procedure. This may be attributed to the fact that cystic tumors often exhibit looser internal structures or the presence of multilocular cysts, complicating the identification of normal anatomical structures and tumor dissection during surgery. Additionally, cystic degeneration may alter the adhesion and delineation between the tumor and surrounding tissues, increasing the difficulty of the surgical operation. Previous literature has also supported the notion that cystic degeneration pose challenges during vestibular schwannoma surgery, thereby prolonging surgical duration and elevating intraoperative risks (3).

Secondly, our findings indicate that compared to SVSs, the CVSs group, while showing no intergroup differences in symptoms at time of diagnosis and DOS, demonstrated a significant decrease in good postoperative facial nerve function and hearing preservation rates. Apart from volumetric factors, the uneven distribution of internal tension within cystic tumors, along with the location of the cystic cavity, may result in more variable patterns of compression on the facial and auditory nerves. Once the cyst wall ruptures or leakage occurs, it can lead to unclear operative fields or difficulties in nerve identification, thereby increasing the risk of postoperative neurological dysfunction. These discoveries emphasize the need for more meticulous imaging assessments and surgical planning in the perioperative period for cystic VSs, while also heightening the team’s awareness of the unique morphological challenges posed by cystic variations.

Furthermore, although univariate analysis suggested that cystic degeneration may be associated with poor facial nerve function and hearing preservation postoperatively, multivariate analysis revealed that the primary independent factors affecting postoperative facial nerve function and hearing preservation are tumor size and patient age. This result aligns with some previous literature, indicating that the increased surgical difficulty attributed to cystic degeneration primarily stems from tumor volume enlargement. While cystic degeneration may exacerbate the tumor’s compression or distortion of cranial nerves to some extent, their ultimate impact on facial and auditory nerve function also depends on multiple factors, including tumor size and the patient’s own neural reserve (8). Therefore, in clinical practice, special attention must be given to nerve protection measures in the surgical planning and intraoperative management of patients with large-volume VSs, regardless of the presence of cystic degeneration.

Finally, it should be noted that this study has limitations due to its retrospective, single-center design, which may introduce selection bias. Additionally, it did not adequately incorporate other potential variables affecting nerve function and surgical risk, such as tumor vascular supply characteristics and types of cystic degeneration. Thus, future multi-center, large-sample prospective studies are necessary to further delineate the role of cystic degeneration in imaging classification and surgical management strategies, providing a more comprehensive evidence base for optimizing the prognosis of patients with CVSs.

In summary, this study preliminarily confirms that CVSs are more likely to lead to prolonged surgical duration and decreased postoperative facial and hearing protection compared to SVSs. However, tumor size and patient age are the primary independent factors influencing postoperative functional prognosis. In clinical decision-making, recognizing and accurately assessing cystic changes is crucial for enhancing perioperative management and individualized treatment strategies.

## 5 Conclusion

The results of this study indicate that there are no significant differences in symptoms at the time of diagnosis, DOS, and GTR rates between CVSs and SVSs. However, the former often requires a longer tumor resection time, and univariate analysis suggests that cystic degeneration may be associated with poorer facial nerve function and hearing preservation. Nonetheless, multivariate analysis reveals that the primary factors affecting postoperative facial nerve function and hearing preservation are tumor volume and age, rather than cystic degeneration. Thus, while cystic degeneration increases the complexity of surgical procedures, their independent impact on prognostic indicators is limited. Clinically, it is essential to emphasize a comprehensive assessment of cystic degeneration and tumor volume, and through precise preoperative planning and individualized surgical strategies, further enhance perioperative management and outcomes.

## Data Availability

The raw data supporting the conclusions of this article will be made available by the authors, without undue reservation.
